# Metabolic flux profiling of recombinant protein secreting *Pichia pastoris* growing on glucose:methanol mixtures

**DOI:** 10.1186/1475-2859-11-57

**Published:** 2012-05-08

**Authors:** Joel Jordà, Paula Jouhten, Elena Cámara, Hannu Maaheimo, Joan Albiol, Pau Ferrer

**Affiliations:** 1 Departament d’Enginyeria Química, Escola d’Enginyeria|, Universitat Autònoma de Barcelona, Bellaterra (Cerdanyola del Vallès), Spain; 2 VTT Technical Research Centre of Finland, Espoo, Finland

## Abstract

**Background:**

The methylotrophic yeast *Pichia pastoris* has emerged as one of the most promising yeast hosts for the production of heterologous proteins. Mixed feeds of methanol and a multicarbon source instead of methanol as sole carbon source have been shown to improve product productivities and alleviate metabolic burden derived from protein production. Nevertheless, systematic quantitative studies on the relationships between the central metabolism and recombinant protein production in *P. pastoris* are still rather limited, particularly when growing this yeast on mixed carbon sources, thus hampering future metabolic network engineering strategies for improved protein production.

**Results:**

The metabolic flux distribution in the central metabolism of *P. pastoris* growing on a mixed feed of glucose and methanol was analyzed by Metabolic Flux Analysis (MFA) using ^13^C-NMR-derived constraints. For this purpose, we defined new flux ratios for methanol assimilation pathways in *P. pastoris* cells growing on glucose:methanol mixtures. By using this experimental approach, the metabolic burden caused by the overexpression and secretion of a *Rhizopus oryzae* lipase (Rol) in *P. pastoris* was further analyzed. This protein has been previously shown to trigger the unfolded protein response in *P. pastoris*. A series of ^13^C-tracer experiments were performed on aerobic chemostat cultivations with a control and two different Rol producing strains growing at a dilution rate of 0.09 h^−1^ using a glucose:methanol 80:20 (w/w) mix as carbon source.

The MFA performed in this study reveals a significant redistristribution of carbon fluxes in the central carbon metabolism when comparing the two recombinant strains *vs* the control strain, reflected in increased glycolytic, TCA cycle and NADH regeneration fluxes, as well as higher methanol dissimilation rates.

**Conclusions:**

Overall, a further ^13^C-based MFA development to characterise the central metabolism of methylotrophic yeasts when growing on mixed methanol:multicarbon sources has been implemented, thus providing a new tool for the investigation of the relationships between central metabolism and protein production. Specifically, the study points at a limited but significant impact of the conformational stress associated to secretion of recombinant proteins on the central metabolism, occurring even at modest production levels.

## Background

*Pichia pastoris* is an attractive system for the production of recombinant proteins [1-4]. Moreover, the development of systems biotechnology tools specific for this cell factory [5-10] has opened new opportunities for strain improvement and rational design of culture conditions.

Several studies have reported on the impact of recombinant protein over expression on different growth parameters of yeast, such as maximum growth rate, biomass yield or substrate specific consumption rate [11-14], suggesting a potential impact on the cell’s central metabolism. Nevertheless, the number of quantitative studies investigating the potential interactions between *P. pastoris*’ central carbon metabolism, environmental conditions and recombinant protein production still remains very limited [9,15-18].

The *AOX1* promoter of *P. pastoris* has been widely used for recombinant protein production. The conceptual basis for this expression system stems from the observation that some of the enzymes required for methanol metabolism are present at substantial levels only when cells are grown on this substrate [19]. Furthermore, catabolite repression by different multicarbon compounds is particularly tight in *P. pastoris*. Interestingly, mixed carbon strategies (mixing methanol with a multicarbon source such as sorbitol or glycerol) have proven to boost productivity levels significantly [2,20], also suggesting that metabolic burden caused by recombinant protein production can be reduced [21].

Overproduction of recombinant proteins may lead to their partial accumulation as misfolded or folding-reluctant protein species within organelles of the secretory pathway, causing considerable stress in the host [22]. This is the case of a *Rhizopus oryzae* lipase (Rol), which has been used as a model protein for several physiological studies of recombinant *P. pastoris*. In particular, over expression of this protein has been shown to trigger the unfolded protein response (UPR) [23], partially explaining its negative effect on cell growth [12]. Even though the unfolded protein response is well characterised in yeast, there are very limited quantitative studies on the potential interactions between an endogenous stress factor (recombinant protein secretion), environmental conditions and the core metabolism.

In this study, biosynthetically directed fractional (BDF) ^13^C-labeling was employed to elucidate the effect of protein burden on the central carbon metabolism of *P. pastoris*. Specifically, this study focuses on comparison between three different strains of *P. pastoris*, two strains producing different amounts of Rol under the control of the *AOX1* promoter (due to different *ROL* gene dosage) and the corresponding control strain. In this way, we aimed at analyzing quantitatively the potential impact (metabolic burden) of recombinant protein secretion on the core and energy metabolism of *P. pastoris*.

## Results and discussion

### Growth and product formation of recombinant *P. pastoris* strains

There are only few physiological studies on the potential impact of recombinant protein production on the core metabolism of *P. pastoris*, particularly for those cases where the product is secreted [9,15]. The aim of our study was to quantify the potential metabolic burden caused by recombinant *R. oryzae* lipase (Rol) secretion on this yeast. This enzyme has proven to be an attractive model for physiological studies of protein production in *P. pastoris*; its over expression triggers the unfolded protein response [23] and it has a negative impact on cell growth parameters, particularly in strains expressing multiple copies of the *ROL* gene [12]. Most notably, Rol secretion levels obtained in *P. pastoris* high cell density cultures are rather moderate (around 300~500 mg L^−1^) [24]. To characterise the potential intracellular carbon flux redistribution due to recombinant Rol secretion, we performed chemostat aerobic cultivations using a fixed mixture of glucose:methanol (80% : 20%, w/w) at a dilution rate of 0.09 h^−1^ for two *P. pastoris* strains producing different amounts of Rol, as well as for the corresponding control strain. The selected dilution rate is below the maximum specific growth rate (μ_max_) of the original Rol-producing strain growing on glucose (0.18 h^−1^) [5], and slightly above the μ_max_ of this strain growing on methanol (0.07 h^−1^) [6], as unique carbon sources. Under these conditions, continuous cultures were carbon-limited, thereby allowing partial glucose derepression of the methanol assimilation pathway, as well as its induction by methanol [25]. Notably, no metabolic by-products could be detected in the culture broth in any of the cultivations. As expected from previous studies, the physiology of *P. pastoris* was affected by Rol overproduction (Table 1). In particular, the specific glucose consumption rate increased significantly from 0.76 to about 0.9 mmol g^−1^ h^−1^ when comparing the reference strain to the two Rol-producing strains, respectively, whereas specific methanol consumption rates were not significantly altered. Furthermore, Rol production also resulted in increased CO_2_ exchange rate (CER) and oxygen uptake rate (OUR) values. Conversely, the physiological differences between the two Rol-producing strains were insignificant, even though extracellular lipase activity levels for the ROL 2-copy strain were about 1.4-fold higher than in the ROL 1-copy strain. Overall, such impact on growth performance is remarkable, since total extracellular protein levels were relatively low (ca. 30 mg L^−1^) in both Rol-producing strains and, furthermore, Rol represented only a fraction (ca. 35–40%) of the total extracellular protein. Therefore, it seemed unlikely that its impact on the physiology of the host could be largely attributed to an increased demand for Rol precursors, but rather as a result of the stress caused to the cell.

**Table 1:** **Growth parameters of *P. pastoris* growing on glucose:methanol in chemostats cultures**

**Strain**	**Glucose mmol/g CDW·h**	**Methanol mmol/g CDW·h**	**OUR mmol/g CDW·h**	**CER mmol/g CDW·h**	**Biomass mmol/g CDW·h**	**Y_X/S_ g CDW/Cmol**	**RQ**	**Lipase activity AU/gCDW**
*X-33 control*	−0.76 ± 0.02	−1.15 ± 0.06	−2.95 ± 0.11	2.31 ± 0.11	3.40 ± 0.04	14.7 ± 0.3	0.78 ± 0.05	n.d.
*ROL 1-copy*	−0.85 ± 0.01	−1.29 ± 0.05	−3.71 ± 0.07	3.04 ± 0.06	3.32 ± 0.03	13.0 ± 0.3	0.82 ± 0.09	2504 ± 192
*ROL 2-copy*	−0.87 ± 0.01	−1.24 ± 0.09	−3.66 ± 0.06	3.04 ± 0.04	3.39 ± 0.05	12.9 ± 0.1	0.83 ± 0.07	3490 ± 208

Reconciled measured substrates and products consumption or production rates at steady state conditions in each experiment. Substrate consumption, biomass and metabolites production rates for each strain. OUR, Oxygen Uptake Rate; CER, CO_2_ Exchange Rate; RQ Respiratory Quotient; n.d., not detectable. Consistency index h was below 7.81 for a redundancy of 3 (95% significance level) in all cases.

Growth conditions and genetic background can have a significant impact on both the elementary and macromolecular composition of cells. Consequently, detailed knowledge of their composition is important for metabolic flux analysis purposes. As there was no such data available for *P. pastoris* growing in glucose:methanol mixtures, determination of elementary composition, protein and carbohydrates (that is, the major cell components, constituting up to 90% of the cell’s dry weight) content, as well as amino acid composition analyses were performed for all of the strains, as shown in Tables 2, 3 and 4 (see also Additional file 1). These analyses revealed that there are significant differences (*p*-value < 0.05) in terms of relative protein content among cultures grown on different carbon sources (glucose *vs*. glucose:methanol), as well as among Rol-producing and control (non-producing) strains. Moreover, several amino acids relative amounts differed significantly (*p*-value < 0.05) when comparing the control strain to the Rol 1-copy strain (Thr and Ala) and to the Rol 2-copy strain (Thr, Ala, Glu, Cys, Met, Orn and Arg) (Table 4). Coherent with the total protein content analyses, the amount of most amino acids was generally higher in cells growing on glucose:methanol than when growing on glucose as a sole carbon source. Following this observation, it was considered that both the carbon source(s) and strain type would have an impact on the calculated metabolic fluxes and, therefore, it was decided to consider a different biomass composition for each of the three strains growing on glucose:methanol (Table 2 and 3).

**Table 2:** **Biomass C-molecular composition for *P. pastoris* **

**A**					
**Strain**	**Carbon source**	**C-mol Biomass formula**	**C:N Ratio**	**H:O Ratio**	**γ**
X-33 control	80% Glucose 20% Methanol	CH_1.687_N_0.17_ O_0.635_S_0.002_	5.9	2.7	3.9
*ROL* 1-copy	80% Glucose 20% Methanol	CH_1.749_N_0.141_ O_0.679_S_0.002_	7.1	2.6	4.0
*ROL* 2-copy	80% Glucose 20% Methanol	CH_1.702_N_0.14_ O_0.643_S_0.002_	7.1	2,6	4.0
X-33 control*	100% Glucose	CH_1.761_N_0.143_ O_0.636_S_0.0018_	7.0	2.8	4.1
S. cerevisiae**	100% Glucose	CH_1.748_N_0.148_ O_0.596_S_0.0018_	6.8	2.9	4.2

*Data taken from [26] ; ** Data taken from [27].

Biomass C-molecular formula for *P. pastoris* growing on glucose:methanol mixture (80:20) as a carbon source in chemostat cultures at a *D* = 0.09 h^−1^, expressed as C-molecular formula. C:N, carbon:nitrogen ratio; H:O, hydrogen:oxygen ratio. γ, reduction degree of the biomass.

**Table 3:** **Biomass macromolecular composition for *P. pastoris* **

**B**					
**Strain**	**Protein w/w**	**Carbohydrate w/w**	Lipids^*^w/w	RNA^*^w/w	DNA^*^w/w
*X-33 control*	0.49 ± 0.02	0.31 ± 0.01	0.02 ± 0.02	0.07 ± 0.007	0.001 ± 0.0001
*ROL 1-copy*	0.43 ± 0.02	0.34 ± 0.01	0.04 ± 0.02	0.06 ± 0.007	0.001 ± 0.0001
*ROL 2-copy*	0.43 ± 0.03	0.34 ± 0.01	0.05 ± 0.02	0.07 ± 0.007	0.001 ± 0.0001

* calculated values.

Biomass macromolecular formula for *P. pastoris* growing on glucose:methanol mixture (80:20) as a carbon source in chemostat cultures at a *D* = 0.09 h^−1^. Data given as percentage of dry cell weight.

**Table 4:** **Amino acid composition of *P. pastoris* **

	**80% Glucose 20% Methanol**			**100% Glucose**	
**% mol/mol**	**X-33**	**ROL 1-copy**	**ROL 2-copy**	X-33*	*S. cerevisiae* **
**Arg**	8.15	7.67	6.45	7.04	3.86
**Asp**	9.25	9.48	10.05	8.78	9.28
**Thr**	6.15	6.06	6.46	5.88	5.57
**Ser**	6.34	6.32	6.82	6.26	5.33
**Glu**	15.76	15.44	13.23	17.81	15.48
**Pro**	4.2	4.0	3.92	3.83	4.22
**Gly**	7.80	7.53	7.86	6.86	8.89
**Ala**	11.14	9.41	9.36	10.40	9.77
**Val**	6.81	6.67	6.54	5.88	7.33
**Cys**	0.17	0.19	0.13	0.15	0.14
**Met**	0.78	0.75	0.62	0.79	1.14
**Ile**	4.49	4.49	4.69	4.64	5.89
**Leu**	7.50	7.45	8.34	6.96	8.01
**Tyr**	2.28	2.19	2.51	2.16	1.96
**Phe**	3.44	3.31	3.61	3.20	3.76
**Orn**	0.68	0.32	0.26	1.04	0.24
**Lys**	6.77	6.77	7.21	6.41	6.57
**His**	2.10	1.94	1.95	1.89	1.93
**Trp**	1.00	1.00	1.00	1.40	1.96

* Data taken from [26]; ** Data taken from [27].

Amino acid composition of the whole cell extract for all the *P. pastoris* strains tested. Amino acid composition measured for the three different *ROL* gene dosages. Data given as percentage of each amino acid in the total pool of amino acids. The standard deviation of the analytical method was 3~5%.

### Impact of methanol co-assimilation on the central carbon metabolism of *P. Pastoris* growing on glucose methanol mixtures

The 2D ^1^H-^13^C-HSQC spectra were analysed as described by [28] and [29], yielding the relative abundances of intact C2 and C3 fragments in proteinogenic amino acids *f*-values (Additional file 2). Analysis of the *f*-values were coherent with the biosynthetic pathways of proteinogenic amino acids in yeast, as already shown in previous studies of *P. pastoris*[5]. The use of the C6 source glucose and C1 source methanol for BDF ^13^C-labelling of proteinogenic amino acids enabled the determination of the flux ratios for reactions associated with the assimilation of C1 source by the cell. When yeast are grown on glucose as a sole carbon source, the *f*-values of His-Cα and Phe-Cα must be equal or practically equal due to the effect of transaldolase and transketolase reactions [15,30]. However, these two patterns were different in our experiments (Additional file 2), providing a direct evidence of assimilation of methanol for cell growth and maintenance, as well as proving that glucose limiting conditions allow for induction of the methanol assimilation pathways by the latter substrate. Similar evidence has also previously been observed in *P. pastoris* cells, growing in glycerol:methanol mixtures, under carbon-limiting conditions [6].

**Figure 1:**
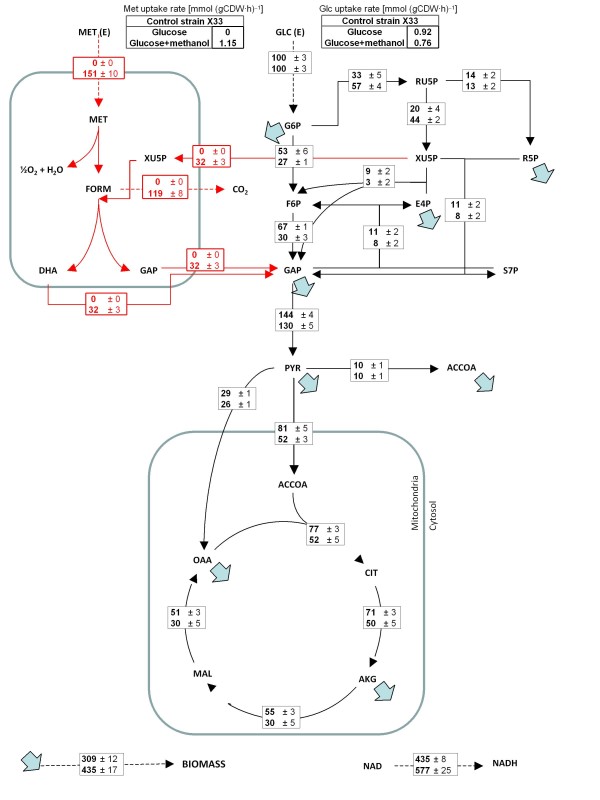
**Metabolic flux distributions in the *P. pastoris* during growth in glucose and glucose:methanol.** Metabolic flux distributions in the *P. pastoris* reference strain during growth in glucose (top) and glucose:methanol (bottom) chemostat cultures at about 0.1 h^−1^. Fluxes are normalized with respect to glucose uptake rate (% C-mol/C-mol glucose). Activities of the malic enzyme and glyoxylate pathways were found to be negligible on the basis of the METAFoR analyses. Metabolic flux data for glucose-grown *P. pastoris* was taken from [10].

When comparing glucose vs glucose:methanol carbon flux distributions in *P. pastoris* cells growing aerobically in carbon-limited chemostat cultures, a clear impact of methanol assimilation is observed on the metabolic network operation (Figure 1). The split ratio between the glycolytic and the oxidative branch of the pentose phosphate pathway (PPP) fluxes was clearly shifted to the latter pathway in glucose:methanol grown cells, probably reflecting the demand of pentose phosphates for methanol assimilation. Also, cells growing in the mixed substrate presented a significantly lower flux through the tricarboxylic acids (TCA) cycle (normalized to the glucose uptake rate). This difference was also reflected in the calculated NADH regeneration rates (Figure 1). Interestingly, Solà and the co-workers [6] did not detect significant differences in the relative TCA cycle activity when comparing cells grown on glycerol vs glycerol:methanol mixtures. As expected from early mixed substrate studies of methylotrophic yeasts [31], methanol co-assimilation resulted in slightly lower biomass yield (14.3 ± 0.3 g CDW/ C-mol) compared with glucose-only grown cultures (16.8 ± 1.2 g CDW/ C-mol). Nevertheless, considering the standard deviations of these calculated values, further experimental data would be required to confirm this tendency. Most notably, most methanol (about 80%) was directly dissimilated to CO_2_, as opposite to cells growing on methanol as sole carbon source under limiting conditions [16]. Methanol co-assimilation has an important effect on the bioenergetics of methylotrophic yeasts. For instance, direct oxidation of methanol to CO_2_ via formaldehyde and formate (see Figure 1) yields two 2 NADH mols per methanol mol. Early studies on methanol metabolism showed that the enzymes for primary oxidation and assimilation of methanol or formaldehyde are under inhibitory control of energy equivalents, e.g. NADH and ATP (formaldehyde and formate dehydrogenases), ADP (dihydroxyacetone kinase) and AMP (fructose-l,6-bisphosphatase) [32]. In methylotrophic yeasts these metabolic pathways operate in accordance with the balance of consumed and produced energy equivalents, thus providing energetic regulation of formaldehyde oxidation and assimilation. Overall, the results seem to reflect the fact that the glucose-only cultivation is NADH-limited and the co-assimilation of methanol as auxiliary substrate may provide an extra direct source of NADH [33].

### Impact of Rol secretion on the central carbon metabolism of *P. pastoris*

As previously observed [12], Rol overproduction had an impact on the substrate specific consumption rate. In addition, both Rol-expressing strains showed slightly lower but significant biomass yields, as well as higher CER and OUR values, compared to the reference strain. This phenomenon might be related to higher energy demand caused by Rol secretion, resulting in higher maintenance-energy requirements. Since Rol amounts were very small relative to the total cell protein, one is tempted to speculate that such metabolic burden was mainly associated to the secretion stress triggered by [Rol 23,^34^], rather than to an increased demand for building blocks. The latter situation seems to occur when recombinant proteins are produced (intracellularly) at high levels in *P. pastoris*[17,18] and other hosts.

The impact of Rol expression on *P. pastoris* core metabolism could be already inferred from the calculated estimated flux ratios (Table 5), reflecting an limited but significant effect of Rol secretion on methanol assimilation, particularly in the ROL 2-copy strain: The relative contribution of methanol to the phospho*enol*pyruvate (Pep) pool was decreased, suggesting that a higher fraction of the assimilated methanol was directly oxidised to CO_2_ (thereby generating 2 mol of NADH per mol of methanol) in the Rol-producing strains.

**Table 5:** Metabolic flux ratio (METAFoR) analysis results

** *% Fraction of total pool* **	**X-33 control**	** *ROL* 1- copy**	** *ROL* 2-copy**	X-33 control*
	*Glucose:Methanol*	*Glucose:Methanol*	*Glucose:Methanol*	*Glucose*
**Pep from methanol**	45 ± 5	34 ± 9	24 ± 9	n.a.
**Pep from PPP (upper bound)**	14 ± 4	21 ± 8	24 ± 11	39 ± 9
R5P from T3P and S7P (transketolase)	77 ± 7	70 ± 1	83 ± 2	66 ± 2
R5P from E4P (transaldolase)	56 ± 1	57 ± 9	56 ± 4	40 ± 2
Ser originating from Gly and C1-unit	53 ± 3	55 ± 2	58 ± 1	62 ± 4
Gly originating from CO_2_ and C1-unit	7 ± 1	12 ± 1	8 ± 2	6 ± 4
Pep originating from Oaa_cyt_ (PepCK)	n.a.	n.a.	n.a.	2 ± 5
**Oaa**_**mit**_**originating from Pep**	49 ± 3	42 ± 12	36 ± 2	n.a.

*Data taken from [26].

METAFoR analysis results showing the origins of metabolic intermediates during growth of *P. pastoris* on glucose:methanol compared with those growing on glucose as the sole carbon source, n.a. (not available). Flux ratios used for MFA are highlighted in bold.

**Figure 2:**
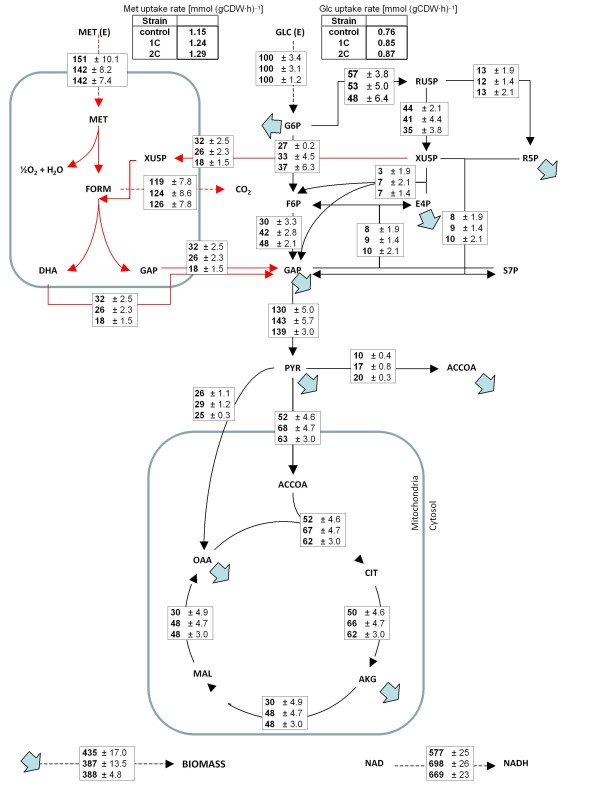
**Metabolic flux distributions in the *P. pastoris* reference and recombinant strains during growth on glucose:methanol.** Metabolic flux distributions in the *P. pastoris* reference strain (top), the recombinant strain with 1 copy of the *ROL* gene (middle) and the recombinant strain harbouring 2 copies of the *ROL* gene (bottom) during growth on glucose:methanol chemostat cultures at about 0.09 h^−1^. Fluxes are normalized with respect glucose uptake flux (% C-mol/C-mol glucose). Activities of the malic enzyme and glyoxylate pathways were found to be negligible on the basis of the METAFoR analyses.

**Figure 3:**
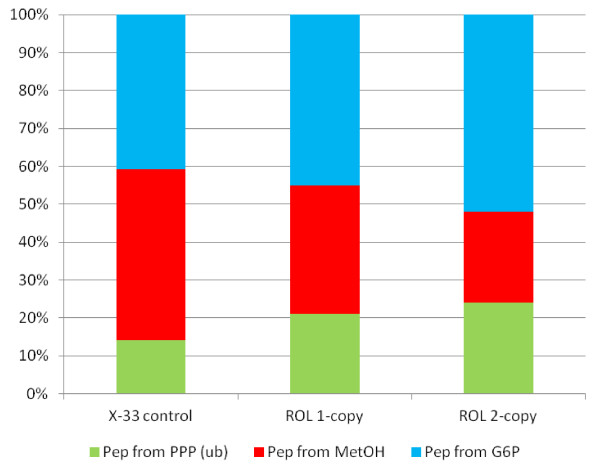
**Fractional distributions of carbon fluxes to phosphoenolpyruvate synthesis derived from**^**13 **^** C-MFA in *P. pastoris.* ** Fractional distributions of carbon fluxes to phosphoenolpyruvate synthesis derived from ^13 ^ C-MFA in *P. pastoris* Rol-producing (*ROL* 1-copy and *ROL* 2-copy) and control (X-33 control) strains growing in glucose-limited chemostats at *D* = 0.09 h^−1^.

Metabolic flux analysis allowed identification of limited but statistically significant changes in the fluxes through central carbon metabolism (Figure 2 and Additional file 3). Since glucose uptake rates were higher in the Rol-producing strains, glycolytic fluxes were concomitantly increased compared to the reference strain. In addition, the flux of pentose phosphates to the methanol assimilation pathway was significantly decreased in Rol-producing strains, consistent with lower fluxes of methanol being assimilated to glyceraldehyde 3-phosphate (GAP), particularly in the ROL 2-copy strain (Figure 3). Notably, although the biomass yield of the Rol-producing strains was somewhat lower compared to the reference strain (also reflected in lower flux to biomass synthesis, Figure 2), the flux through the oxidative branch of the PPP appeared to be similar in all strains. Since one would expect a correlation between the biomass yield and the activity of PPP [35], such effect could be the result of an increased demand of reduction equivalents (NADPH) to regenerate reduced glutathione (GSH) in the endoplasmatic reticulum (ER), the electron donor in the protein folding oxidative process [36,37]. Interestingly, direct methanol dissimilation to CO_2_ also involves an oxidative step using GSH as electron donor.

Further evidence for a metabolic burden derived from Rol expression was indicated by a significantly increased relative flux through the TCA cycle (normalized to the glucose uptake rate) in the Rol-producing strains. In addition, a tendency to increase the flux through the methanol dissimilatory pathway to CO_2_ was observed in the Rol-producing strains compared to the reference strain, also reflected in a slight increase in the split ratio between this pathway and the assimilatory pathway to Pep; however, such tendency was not statistically significant. Coherent with these two observations, the respiration rate increased (see CER, OUR and respiratory quotient (RQ) values in Table 1), reflecting a significant increase in NADH regeneration reactions and, probably, in ATP generation (Figure 2). This would suggest that the metabolic burden caused by Rol secretion is at least partially compensated by increased energy production. That is, the Rol-producing strains appear to have higher maintenance requirements compared to the control strain. Since Rol production levels are moderate, the correlation observed between the specific Rol secretion rates and NADH regeneration rates provide indirect evidence on the metabolic burden associated with protein folding and conformational stress. Interestingly, Heyland and co-workers [17] observed a similar effect (increased TCA cycle flux of 1.1 mmol g CDW h^−1^ in the producing strain compared to 0.7 mmol g CDW h^−1^ in the reference strain) in *P. pastoris* cells producing an intracellular recombinant protein growing on glucose in a fed-batch culture at a controlled growth rate of 0.12 h^−1^. However, in a recent study by the same group [18], where a series of *P. pastoris* strains expressing different levels of a model recombinant protein exponentially growing in shake cultures (that is, at maximum specific growth rate) were compared, revealed that although there was a relative increase in the relative TCA cycle activity in the producing strains compared to the reference strain, the absolute TCA cycle activity remained constant around 2.1 ± 0.1 mmol g CDW h^−1^ in all strains, suggesting an upper limit of TCA cycle activity and, thereby postulating that cells do not have the capacity to catabolize a sufficient amount of carbon through the TCA cycle to fully compensate the higher energy demand derived from recombinant protein overproduction. In the present case, the value of the TCA cycle activity was 0.39 ± 0.03 mmol gCDW h^−1^, 0.55 ± 0.03 mmol gCDW h^−1^, and 0.57 ± 0.04 mmol gCDW h^−1^ for the control strain, single and 2-copy Rol-producing strains, respectively, clearly below the reported hypothetical activity upper TCA cycle limit for *P. pastoris* growing aerobically on glucose [18]. Furthermore, since there is a significant increase in the absolute flux through the TCA cycle in Rol-producing strains, it seems plausible that there is no energetic limitation at this level in cells growing on glucose:methanol mixtures. In fact, co-assimilation of methanol as auxiliary substrate might be a mechanism by which the increased energy demand of the producing strains is compensated.

## Methods

### Strains and media

A series of recombinant *P. pastoris* X-33 (Invitrogen) derived strains were used in this study. Namely, a control strain harbouring pGAPAα (Invitrogen) as mock plasmid [26], and two different Rol producing strains: i) the X-33/pPICZAα-ROL strain, previously obtained by [24], regarded as a strain containing a single copy of the *ROL* expression vector integrated at the host’s *AOX1* genomic locus and, ii) a strain newly generated in this study following a second transformation of strain X-33 with pPICZAα-ROL using a electroporation procedure described by [38]. Prior to transformation, plasmid DNA was linearized to promote integration at the *AOX1* locus. Transformants were selected on YPD agar plates containing 100 mg L^−1^ zeocin (Invivogen) and were subsequently replica-plated onto selection YPD agar plates containing 1000 mg L^−1^ zeocin, as a strategy to select transformants containing multiple copies of the *Rol* expression vector integrated in their genome [12,38,39]. To select a multi-copy strain with higher expression levels, 10 independent transformants from agar plates containing 1000 mg L^−1^ zeocin were tested for extracellular lipolytic activity in 500 mL Erlenmeyers following standard procedures described in the *Pichia Expression Kit Manual* (Invitrogen). The best clone, was further selected for chemostat studies. Both single and multicopy Rol-producing strains were further characterised in terms of *ROL* gene dosage by quantitative real-time PCR.

### qRT-PCR assay

Quantitative real-time PCR was carried out in 20 μL volume reactions using semi-skirted iQ 96-well PCR plates and SsoFast^TM^ EvaGreen® Supermix (both from Bio-Rad). Samples were measured in triplicate and standards were measured in duplicate on the iCycler Thermal Cycler (Bio-Rad). A non template control was run in every experiment for each of the primer pairs to avoid detection of unspecific priming. The reactions were incubated at 95°C for 5 min to activate the Taq polymerase, and then subjected to a three-step cycling protocol including melting (94°C, 15 s), annealing (58°C, 15 s) and extension (72°C, 30 s) for a total of 40 cycles. Each extension was followed by data collection at 72°C and a short incubation step at 78°C (1 s) for a second plate reading closer to the melting point. Following a final extension of 5 min at 72°C, we generated a melting-curve profile collecting data along 70 cycles with variable temperature starting at 60°C, with 0.5°C increments/cycle (1-s intervals). The primers used for the amplification reaction were 5′ CCCTGTCGTCCAAGAACAAC 3′ and 5′ GAGGACCACCAACAGTGAAG 3′ (forward and reverse primers, respectively) for the *ROL* gene; for the reference amplification reaction of the β-actin gene (*ACT1*), primers were the same as described previously in [15]. The relative gene expression level was calculated for each sample in triplicate measurements giving a maximum standard deviation around 10%. Since the amplification efficiencies of the target and reference genes were not the same in our experiments, we used the Pfaffl method [40] for the relative quantification of our qRT-PCR results.

### Chemostat cultivations

Two duplicate aerobic, carbon-limited continuous cultures for each of the three strains were carried out in a 3 L vessel bioreactor (Applikon Biotechnology) that was controlled at 25°C. The working volume was kept at 1 L by means of an overflow system. The pH was controlled at 5.0 with 1 M NH_3_. An aeration rate of 1 vvm, controlled by the bioreactor’s mass flow meters, and a stirring rate of 800 rpm allowed maintaining dissolved oxygen levels at a minimum of 15% of air saturation. An overpressure of 0.2 bar was applied to the system to facilitate sampling of broth. The chemostat cultures were set at a *D* of 0.09 h^−1^ by feeding a defined growth medium [41] containing 50 g L^−1^ of glucose/methanol mixture (80% glucose / 20% methanol, w/w) as a carbon source. The bioreactor off-gas was cooled in a condenser (4°C), dried by means of two silica gel columns and subsequently analyzed with BCP-CO_2_ and BCP-O_2_ sensors (Blue-Sens). Sensors were calibrated using a series of 3 calibration gases containing CO_2_/O_2_/N_2_ mixtures in the following percentages, respectively: 1/20.9/78.042; 3/5/91.97; 7/ 0/93. Steady state samples were taken after the cultures had been in constant conditions for a minimum of five residence times. Steady states were assessed over 4 to 6.5 residence times for constant biomass production CER, OUR, and detectable extracellular metabolites.

### Analytical procedures

*Biomass analyses.* The cell concentration was monitored by measuring the optical density of cultures at 600 nm (OD_600_). For cell dry weight (CDW) measurement, 5 mL of culture broth was filtered using pre-weighed dried glass fiber filters (Millipore). Cells were washed twice using the same volume of distilled water and dried overnight at 100°C. Triplicate samples (5 mL) were taken for all optical density and cell dry weight measurements. Biomass samples for the determination of the elemental composition, as well as amino acid, total protein and carbohydrate contents were prepared and analyzed as described by [26]. The measured amino acid content of the biomass allowed on the one hand, estimating the total protein content of the biomass and, on the other hand, calculating a specific protein composition to be used in a synthesis equation for the metabolic flux calculations. The experimentally measured elemental components (C, H, N, S and ash content). Oxygen content was calculated by difference as the remaining component. Major macromolecular biomass components (proteins and carbohydrates) were reconciled as previously described [27]. DNA, RNA and lipid content considered in this data consistency analysis were taken from previous measurements [26]. The resulting balanced biomass macromolecular composition was subsequently used for ^13^ C-constrained metabolic flux analysis. In all chemostat cultivations, the C recovery data was above 92% before applying a data consistency and reconciliation step. The experimental data was verified using standard data consistency and reconciliation procedures [42-44], under the constraint that the elemental conservation relations were satisfied. For all chemostat cultivations performed, the statistical consistency test carried out with a confidence level of 95% was acceptable, and consequently accepting that there was no proof for gross measurement errors.

*Quantification of extracellular metabolites*. Triplicate samples (5 mL) for extracellular metabolite analyses were centrifuged at 6,000 rpm for 3 min in a micro centrifuge (Minispin, Eppendorf) to remove the cells, and subsequently filtered through 0.45 mm-filters (Millipore type HAWP). Glucose, methanol, and other potential extracellular compounds were analyzed by HPLC (Dionex Ultimate 3000) analysis using an ionic exchange column, (ICSep ICE-COREGEL 87 H3, Transgenomic). The mobile phase was 6 mM sulphuric acid. The injection volume was 20 μL and the chromatogram was quantified with the CROMELEON software (Dionex).

*Phosphoenolpyruvate carboxykinase (PepCK) assay*. The activity of PepCK was assayed following a method previously described by [45], using the soluble fraction of cell crude extracts obtained after mechanical disruption using glass beads, as described by [46]. Briefly, cell extracts were prepared by mechanical disruption of 15–20 mg of lyophilized cells resuspended in 0.5 mL of extraction buffer (20 mM Hepes, pH 7.1, 1 mM DTT, 100 mM KCl), Complete protease inhibitor cocktail (Roche) and 1 g of glass beads. Cell suspensions were subsequently vortexed for 6 periods of 30 s, with a 30 s interval in ice between each vortexing cycle. Samples were centrifuged at 3000 ×g for 5 min at 4°C and the supernatant was subsequently transferred into an Eppendorf tube. A final centrifugation step was carried out in Eppendorf tubes at 5000 ×g for 15 min and 4°C to ensure that the final supernatant was totally clear. The resulting supernatant was used as cell-free extract*.* PepCK activity was determined following the 340 nm absorbance of the reduced pyridine nucleotide cofactor (E_340nm_ = 6.22 mM^−1^). The reaction mixture (1 mL) contained 100 μmol Imidazole-HCl buffer, pH 6.6, 50 μmol NaHCO_3_, 2 μmol MnCl_2_, 2 μmol reduced glutathione, 2.5 μmol ADP, 0.15 μmol NADH, 3 U of malate dehydrogenase (Roche), and cell-free extract, 10~50 μL. The reaction was started by adding 2.5 μmol Pep. Enzyme activity was measured in a spectrophotometer (Varian Cary 300) at 30°C and 340 nm [45]. One unit activity is defined as the amount of enzyme that catalyzes the formation of 1 μmol of reduced pyridine nucleotide per min.

*Lipase activity assay*. The lipolytic activity was performed as previously described in [47].

### Biosynthetically directed fractional (BDF) ^13^ C-labelling

*P. pastoris* cells were fed with a minimal medium containing 50 g L^−1^ of a glucose:methanol mixture (80% glucose / 20% methanol, w/w) for five bioreactor volume changes until reaching a metabolic steady state, as indicated by a constant cell density in the bioreactor and constant O_2_ and CO_2_ concentrations in the exhaust gas. The ^13^C-labelling experiments were performed in two replicate cultures for each strain.

BDF ^13^C labelling of cells growing at steady state on a mix of two carbon source has been described elsewhere [6]. Briefly, as two carbon sources (namely, glucose and methanol) were used, the BDF ^13^C labelling step involved feeding the reactor with the medium containing about 12% (w/w) of uniformly ^13^C-labelled and 88% unlabelled amounts of each substrate simultaneously fed for 1.5 volume changes. [U-^13^C] glucose (isotopic enrichment 99%) and ^13^C-methanol (isotopic enrichment 99%) were purchased from Cortecnet (Voisins le Bretonneux, France). The labelled substrates were fed for a period of 1.5 residence times, after which, a volume of about 500 mL of culture broth was harvested, centrifuged at 4000 ×g for 10 min, resuspended in 20 mM Tris·HCl, pH 7.6, and centrifuged again. The recovered and washed cell pellets were freeze dried (Benchtop 5 L Vitris Sentry, Virtis Co., Gardiner, NY, USA). Finally, 100 mg of the freeze dried cell pellets were suspended into 10 mL of 6 M HCl and the biomass was hydrolysed in sealed glass tubes at 110°C for 22 h. The suspensions were dried overnight in an oven at 90°C, dissolved in H_2_O and filtered through 0.2 μm filters (Millipore). The filtrates were vacuum-dried and dissolved in D_2_O for NMR experiments. The final pH of the samples was below 1 due to residual HCl.

### NMR spectroscopy

^1^H-^13^C-HSQC nuclear magnetic resonance (NMR) spectra of the samples were acquired at 40°C on a Varian Inova spectrometer operating at a ^1^H-resonance frequency of 600 MHz essentially as described in [28]. For each sample two spectra focusing on the aliphatic and aromatic regions were acquired as previously reported in [28]. The spectra were processed using the standard Varian spectrometer software VNMR (version 6.1, C).

### Metabolic flux ratio (METAFoR) analysis

The software FCAL (R.W. Glaser; FCAL 2.3.1) [29] was used for the integration of ^13^C-scalar fine structures of proteinogenic amino acid carbon signals in the ^1^H-^13^C-HSQC NMR spectra and for the calculation of relative abundances of intact carbon fragments originating from a single molecule of glucose. The nomenclature used here for the intact carbon fragments, fragmentomers, has also been described previously [48]. Briefly, *f*^(1)^ represents the fraction of molecules in which the observed carbon atom and the two neighbouring carbons originate from different carbon source molecules (glucose and methanol), *f*^(2)^ the fraction of molecules in which the observed carbon atom and one of the two neighbouring carbon atoms originate from the same source molecule of glucose, as methanol is a single carbon compound, and *f*^(3)^ represents the fraction of molecules in which the observed carbon atom and both carbon neighbours originate from the same glucose molecule. In case that the observed carbon exhibits significantly different ^13^C-^13^C scalar coupling constants for the two neighbour carbons, two different fractions, *f*^(2)^ and *f*^(2*)^ are distinguished. In this case, the fraction of molecules with a conserved bond between the observed carbon atom and the neighbouring carbon with the smaller coupling is represented by *f*^(2)^. Accordingly, *f*^(2*)^ then denotes the fraction of molecules where the carbon bond is conserved between the observed carbon and the neighbouring carbon with the larger coupling. If the observed carbon is located at the end of a carbon chain, only the *f*^(1)^ and *f*^(2)^ fragmentomers can be observed. The fragmentomer information obtained from the proteinogenic amino acids can be traced back to their metabolic precursors, which are intermediates of central carbon metabolism. The carbon backbones of those eight precursors are conserved in the amino acid synthesis pathways [30]. The compartmentalized metabolic network considered for the METAFoR analysis of *P. pastoris* growing on glucose/methanol mixtures was described in [6]. Due to the assimilation of two different carbon sources, the flux ratio calculation methodology for eukaryotic cells described in [6] was further extended as follows: The fraction of phospho*enol*pyruvate (Pep) originating from phospho*enol*pyruvate carboxykinase activity was assumed negligible on the basis that this “gluconeogenic” enzyme is not involved in methanol assimilation [19] and its activity appeared to be negligible in *P. pastoris* cells growing under the experimental conditions chosen in this study. Consequently, Pep was considered to originate from three sources: the Pentose Phosphate Pathway (PPP), glycolysis and, the methanol assimilation pathway (Equation 1).

(1) X1+X2+X3+X4=Pep

Where X1 and X2 are the relative fluxes from glyceraldehyde phosphate (GAP) and dihydroxyacetone phosphate (DHAP) to Pep, respectively, both derived from the methanol assimilation pathway, X3 the relative flux from glycolysis to Pep, and X4 is the relative flux from the PPP to Pep. Glycolysis produces fully intact three carbon fragments into Pep molecules [28], whereas the Pep molecules originating from PPP and the methanol assimilation pathway are partially cleaved. Furthermore, the Pep molecules originating from the methanol assimilation pathway possess partially different labelling patterns than the Pep molecules originating from PPP. The contribution of the PPP pathway results in the interconversion of three pentose phosphate molecules to five molecules of Pep. Among the Pep molecules originating from PPP, three fifths retain the C3-C4-C5 fragment of the pentose phosphates, while two fifths possess the C1-C2 fragment of the pentose phosphates and a single newly formed C-C bond [28]. The methanol assimilation pathway carries the equal pentose phosphate fragments to Pep as PPP but also reversed C3-C4-C5 pentose phosphate fragments since DHAP can reverse the orientation. The methanol is assimilated by forming a six carbon molecule which is then cleaved into two three-carbon compounds, GAP and DHAP. DHAP was assumed to react fully symmetrically. The Pep molecules originating from the reversed DHAP molecules possess different labelling patterns than the Pep molecules originating from PPP. The fraction of Pep originating from the different metabolic pathways was derived from mass balances of the Pep C2 fragmentomers *f*^(2)^ and *f*^(2^*^)^, whereby back-tracking the amino acid synthesis pathways of Phe-Cα and Tyr-Cα to the C2 of Pep [28]. Taking all the above considerations into account the following mass balances were derived from the metabolic reaction carbon mappings of ARM [49] database for the *f*^(2)^ and *f*^(2*)^ fragmentomers of Phe-Cα and Tyr-Cα:

(2) $$f^{\left(2\right)}\left\{Phe,Tyr-C\alpha \right\}=X1\cdot f^{\left(2\right)}\left\{His-C\delta \right\}\cdot 0.5+X2\cdot f^{\left(2*\right)}\left\{His-C\alpha \right\}+X4\cdot \left(\frac{2}{5}\cdot f^{\left(2\right)}\left\{His-C\delta \right\}+\frac{3}{5}\cdot f^{\left(2*\right)}\left\{His-C\alpha \right\}\right)$$

(3) $$f^{\left(2*\right)}\{Phe,Tyr-C\alpha \}=X1\cdot f^{\left(2\right)}\left\{His-C\delta \right\}\cdot 0.5+X2\cdot f^{\left(2*\right)}\left\{His-C\alpha \right\}+X4\cdot \frac{3}{5}\cdot f^{\left(2\right)}\left\{His-C\alpha \right\}$$

Taking into account equations (1), (2) and (3), we have formulated the relative contributions of the different pathways to the synthesis of Pep. The relative flux from the methanol assimilation pathway to Pep (XPep_from_MetOH) is a sum of the equal contributions X1 and X2 from glyceraldehyde phosphate (GAP) and dihydroxyacetone phosphate (DHAP), respectively (Equation 4). The relative flux from PPP (X4) is named XPep_from_PPP below (Equation 5). The contribution of Glycolysis to the synthesis of Pep (X3) was solved from Equation 1.

(4) $$X_{Pep\_from\_MethOh}=\frac{f^{\left(2\right)}\left\{Phe,Tyr-C\alpha \right\}-X4\cdot \left(\frac{2}{5}\cdot f^{\left(2\right)}\cdot \left\{His-C\delta \right\}\right)+\frac{3}{5}\cdot f^{\left(2*\right)}\left\{His-C\alpha \right\}}{f^{\left(2\right)}\left\{His-C\delta \right\}0.5+f^{\left(2*\right)}\left\{His-C\delta \right\}}$$

(5) XPep_from_PPP=f(2*)Phe,Tyr−Cα−X1·f(2)His−Cδ0.5−X2·f(2)His−Cδ35·f(2)His−Cα

The fraction of mitochondrial oxaloacetate (Oaa_mit_) originating from Pyr_cyt_ through pyruvate carboxylase denoted by X_Oaamit___from___Pyrcyt_, was derived from the mass balance of intact C2-C3 fragments of Oaa (Equation 4). The Oaa molecules originating from the TCA cycle are fully cleaved in C2-C3. Since the flux from Pep to Pyr_cyt_ is known to be unidirectional under the carbon-limited cultivation conditions studied here, the Phe-Cα and Tyr-Cα fragmentomers having Pep as a precursor were used to represent the labelling status of Pyr_cyt_. Oaa is the precursor of Asp and Thr and the carbon backbone of Oaa_mit_ in particular is conserved also in the TCA cycle and can be observed in Glu. The Asp, Thr and Glu labelling patterns showed an equal cleavage status as their precursor Oaa. Thus, Asp-Cα and Thr-Cα fragmentomers were used here to represent Oaa_mit_ C2.

(6) $$X_{Oaa_{\mathrm{mit}}\_from\_Pyr_{\mathrm{cyt}}}=\frac{\left(f^{\left(3\right)}+f^{\left(2\right)}\right)\cdot \left\{Asp,Thr-C\alpha \right\}}{\left(f^{\left(3\right)}+f^{\left(2\right)}\right)\cdot \left\{Phe,Tyr-C\alpha \right\}}$$

As described previously [5,28-30], the calculation of metabolic flux ratios when using fractional ^13^C-labeling of amino acids is based on assuming both a metabolic (see above) and an isotopomeric steady state. As stated above, to establish a cost-effective protocol for a larger number of ^13^C labelling experiments, we fed a chemostat operating in metabolic steady state for the duration of 1.5 volume changes with the medium containing the ^13^C-labelled substrates before harvesting the biomass. Then, the fraction of unlabeled biomass produced prior to the start of the supply with ^13^C-labelled medium can be calculated following simple wash-out kinetics [5].

### ^13^C-metabolic flux analysis

^13^C-constrained metabolic flux analysis (^13^C-MFA) was performed using a stoichiometric model comprising the major pathways of *P. pastoris* central carbon metabolism. To calculate the intracellular net fluxes, the model was constrained with extracellular flux parameters (evolution rates of biomass, methanol and glucose uptake rate, CO_2_ uptake rate) and 3 intracellular ratios derived from the METAFoR analysis (see Table 5), as described by [50], thereby constituting a determined system. Therefore, redox cofactors were not used as mass balance constraints to solve the ^13^C-MFA system. Cofactor mass balances are potential sources of errors since the correct balancing requires detailed knowledge of the relative activities of different isoenzymes and the enzyme cofactor specificities on a cell wide scale. Error minimization for the flux calculations in the determined network was carried out as described by [15]. The stoichiometric model of central carbon metabolism of *P. pastoris* was formulated following the model utilized by [15], complemented with the methanol assimilation pathways (Additional file 4). Glyoxylate cycle and malic enzyme reaction were omitted from the model on the grounds of the inspection of the METAFoR analysis, as previously described [48]. In this model, the consumption of central metabolic pathway’s intermediate metabolites for formation of the major biomass macromolecular components (proteins, carbohydrates, lipids and nucleic acids), was calculated as previously described [26] and considering *P. pastoris* biosynthetic pathways [5,6,51,52]. The metabolic fluxes were considered as net fluxes so that a net flux in the forward direction was assigned a positive value and a net flux in the reverse direction was assigned a negative value.

### Calculation of NADH regeneration rates

The rate of NADH regeneration was derived from the determined fluxes. Once a solution of the metabolic system was found, the metabolic fluxes were used to perform a theoretical calculation of the oxygen consumed. For this purpose, all major steps involved in oxygen consumption were taken into account (essentially, methanol and lipid biosynthesis pathways, as well as all relevant electron balances). Furthermore, it was assumed that all NADPH generated was consumed in biosynthetic reactions. Therefore, all the remaining reduction equivalents were assumed to be recycled through the respiratory chain as any other relevant possibility for recycling has already been taken into account. This allowed calculating the theoretical oxygen consumption rates. The theoretical oxygen consumption rates calculated represented 92% of the experimentally measured ones. Those results indicate that under the tested experimental conditions, the calculated variables are highly consistent with the experimental ones.

### Statistical analyses

Data are given as mean ± SEM. Where appropriate, values were compared by a t-test, and significant differences were considered if above a 95% confidence level (*p* < 0.05).

## Conclusions

Overall, the methodology for MFA based on NMR derived ^13^C constraints has been extended to the methanol metabolism, thereby enabling the metabolic analysis of recombinant *P. pastoris* growing on substrate mixtures containing methanol. This methodology, which has also been validated by ^13^C-MFA based on GC-MS data (unpublished results, manuscript in preparation) allowed for the quantitative analysis of the additional energy requirements derived from cell’s adaptation to stress caused by recombinant protein secretion. Importantly, a limited but significant impact on the energy metabolism could be detected even at relatively low secretion levels when comparing the reference strain with the Rol-producing strains, suggesting that protein folding and conformational stress imposes a burden on the central metabolism. Therefore, it points at the core/energy metabolism as an important target for improvement of recombinant protein production processes in yeast, e.g. by engineering new strains with reduced maintenance requirements, more efficient mechanisms of energy generation or by designing new/improved cultivation processes. Nevertheless, metabolic differences between ROL 1-copy and 2-copy producing strains were not statistically significant, suggesting that larger differences in expression/secretion levels are needed in order to have a detectable impact on the central metabolism. Notably, methanol seems to play a key role as auxiliary substrate to compensate for the increased energy demands derived from recombinant protein secretion and favouring metabolic adaptation to the new requirements. This observation could be the underlying explanation why mixed substrate feeding strategies can boost productivities (and reduce metabolic burden) in *P. pastoris*.

## Competing interests

The authors declare that they have no competing interests

## Authors’ contributions

JJ performed bioreactor cultivations and ^13^C-labelling experiments, macroscopic data processing and metabolic flux analysis calculations. PJ and HM, together with JJ, performed the 2D-NMR and METAFoR analyses (including the formulation of new metabolic flux ratios for methanol metabolism), as well as the subsequent interpretation of results. EC constructed the ROL 2-copy strain and performed quantitative real-time PCR for gene dosage quantification. JA designed the ^13^C-constrained MFA approach, and participated in analysis and interpretation of MFA results, as well as in the overall conceptual and experimental design of this study. PF participated in the overall conceptual and experimental design, interpretation of results and drafted the manuscript. All authors read and approved the final manuscript.

## Supplementary Material

Additional file 1 **Biomass macromolecular composition for *P. pastoris.* ** Macromolecular formula for the reference and two Rol-producing *P. pastoris* strains growing on glucose:methanol mixture (80:20) as a carbon source in chemostat cultures at a *D* = 0.09 h^−1^, expressed as C-molecular formula. Click here for file

Additional file 2 **Relative abundances of intact carbon fragments in proteinogenic amino acids. **Relative abundances of intact C2 and C3 fragments (*f*-values) in proteinogenic amino acids describing the conservation of carbon chain fragments in *P. pastoris* Rol-producing and control strains growing in glucose:methanol-limited chemostats at D = 0.09 h^−1^. Click here for file

Additional file 3 **Metabolic flux distributions in the *P. pastoris* reference and recombinant strains during growth on glucose:methanol.** Metabolic flux distributions in the *P. pastoris* reference strain (top), the recombinant strain with 1 copy of the *ROL* gene (middle) and the recombinant strain harbouring 2 copies of the *ROL* gene (bottom) during growth on glucose:methanol chemostat cultures at about 0.09 h^−1^. Activities of the malic enzyme and glyoxylate pathways were found to be negligible on the basis of the METAFoR analyses. Click here for file

Additional file 4 **Stoichiometric model of the central carbon metabolism of *P. pastoris* .** Reactions in the stoichiometric model of the central carbon metabolism of *P. pastoris* applied in the ^13^C-MFA determination of the metabolic fluxes under different oxygenation conditions; it also includes anabolic reactions from metabolic intermediates to biosynthesis, transport reactions across the mitochondrial membrane and uptake and excretion reactions. Note that O_2_, CO_2_, energy and redox cofactor mass balances were not included in the mass balance constraints in ^13^C-MFA. Click here for file
