# Supplementary material for: Metabolic flux profiling of recombinant protein secreting Pichia pastoris growing on glucose:methanol mixtures
Source: Microb Cell Fact. 2012 May 8;11:57. doi: 10.1186/1475-2859-11-57 (PMC3443025; doi:10.1186/1475-2859-11-57)
Supplement: Additional file 1 — Biomass macromolecular composition for P. pastoris. Macromolecular formula for the reference and two Rol-producing P. pastoris strains growing on glucose:methanol mixture (80:20) as a carbon source in chemostat cultures at a D = 0.09 h−1, expressed as C-molecular formula. [file 1475-2859-11-57-S1.doc]

**Supplementary file 1. Biomass macromolecular composition for *P. pastoris***

Macromolecular formula for the reference and two Rol-producing *P. pastoris* strains growing on glucose:methanol mixture (80:20) as a carbon source in chemostat cultures at a *D* = 0.09 h^−1^, expressed as C-molecular formula.

| **Strain** | **Protein** Cmol/g CDW | **Carbohydrate** Cmol/g CDW | **Lipids^*^**  Cmol/g CDW | **RNA^*^**  Cmol/g CDW | **DNA^*^**  Cmol/g CDW |
| --- | --- | --- | --- | --- | --- |
| *X-33 control* | 0.61 ± 0.01 | 0.31 ± 0.01 | 0.03 ± 0.03 | 0.05 ± 0.006 | 0.001 ± 0.0001 |
| *ROL 1-copy* | 0.52 ± 0.03 | 0.35 ± 0.02 | 0.08 ± 0.04 | 0.06 ± 0.003 | 0.001 ± 0.0001 |
| *ROL 2-copy* | 0.51 ± 0.03 | 0.34 ± 0.02 | 0.09 ± 0.05 | 0.05 ± 0.003 | 0.001 ± 0.0001 |

* calculated values
