# Supplementary material for: Metabolic flux profiling of recombinant protein secreting Pichia pastoris growing on glucose:methanol mixtures
Source: Microb Cell Fact. 2012 May 8;11:57. doi: 10.1186/1475-2859-11-57 (PMC3443025; doi:10.1186/1475-2859-11-57)
Supplement: Additional file 2 — Relative abundances of intact carbon fragments in proteinogenic amino acids. Relative abundances of intact C2 and C3 fragments (f-values) in proteinogenic amino acids describing the conservation of carbon chain fragments in P. pastoris Rol-producing and control strains growing in glucose:methanol-limited chemostats at D = 0.09 h−1. [file 1475-2859-11-57-S2.doc]

**Supplementary file 2. Relative abundances of intact C2 and C3 fragments in proteinogenic amino acids.**

**Table 1 - First series of labeling experiments**

| ***Strain*** | | | | | | | | | | | | | | |  |
| --- | --- | --- | --- | --- | --- | --- | --- | --- | --- | --- | --- | --- | --- | --- | --- |
|  | X-33 control | | | |  | X-33 *ROL* 1-copy | | | |  | X-33 *ROL 2*-copy | | | |  |
| C atom | *f^(1)^* | *f^(2)^* | *f^(2*)^* | *f^(3)^* |  | *f^(1)^* | *f^(2)^* | *f^(2*)^* | *f^(3)^* |  | *f^(1)^* | *f^(2)^* | *f^(2*)^* | *f^(3)^* |  |
|  |  |  |  |  |  |  |  |  |  |  |  |  |  |  |  |
| Ala-Cα | 0.224 | 0.153 | 0.104 | 0.518 |  | 0.211 | 0.148 | 0.069 | 0.572 |  | 0.23 | 0.137 | 0.072 | 0.561 |  |
| Ala-Cβ | 0.356 | 0.644 | - | - |  | 0.31 | 0.69 | - | - |  | 0.313 | 0.687 | - | - |  |
| Arg-Cβ | 0.673 | 0.327 | - | 0 |  | 0.646 | 0.36 | - | 0 |  | 0.723 | 0.254 | - | 0 |  |
| Arg-Cδ | 0.401 | 0.599 | - | - |  | 0.329 | 0.671 | - | - |  | 0.315 | 0.685 | - | - |  |
| Asp-Cα | 0.46 | 0.146 | 0.272 | 0.133 |  | 0.41 | 0.156 | 0.289 | 0.146 |  | 0.445 | 0.125 | 0.291 | 0.14 |  |
| Asp-Cβ | 0.502 | 0.224 | 0.204 | 0.070 |  | 0.429 | 0.227 | 0.254 | 0.09 |  | 0.465 | 0.183 | 0.278 | 0.07 |  |
| Glu-Cα | 0.459 | 0.208 | 0.252 | 0.08 |  | 0.468 | 0.192 | 0.303 | 0.036 |  | 0.459 | 0.189 | 0.279 | 0.073 |  |
| Glu-Cβ | 0.729 | 0.271 | - | 0 |  | 0.67 | 0.329 | - | 0 |  | 0.736 | 0.34 | - | 0 |  |
| Glu-Cγ | 0.389 | 0 | 0.611 | 0 |  | 0.289 | 0.009 | 0.701 | 0 |  | 0.288 | 0 | 0.721 | 0 |  |
| Gly-Cα | 0.465 | 0.535 | - | - |  | 0.45 | 0.55 | - | - |  | 0.42 | 0.58 | - | - |  |
| His-Cα | 0.174 | 0.077 | 0.074 | 0.674 |  | 0.19 | 0.045 | 0.036 | 0.728 |  | 0.224 | 0 | 0.065 | 0.739 |  |
| His-Cβ | 0.371 | 0.449 | 0 | 0.181 |  | 0.259 | 0.439 | 0.001 | 0.301 |  | 0.352 | 0.492 | 0 | 0.19 |  |
| His-Cδ^2^ | 0.571 | 0.429 | - | - |  | 0.508 | 0.492 | - | - |  | 0.538 | 0.462 | - | - |  |
| Ile-Cα | 0.623 | 0 | 0.377 | 0 |  | 0.572 | 0 | 0.428 | 0 |  | 0.604 | 0 | 0.4 | 0 |  |
| Ile-Cγ^1^ | 0.733 | 0.267 | - | 0 |  | 0.574 | 0.354 | - | 0 |  | 0.734 | 0.302 | - | 0 |  |
| Ile-Cγ^2^ | 0.425 | 0.575 | - | - |  | 0.313 | 0.687 | - | - |  | 0.33 | 0.67 | - | - |  |
| Ile-Cδ | 0.761 | 0.239 | - | - |  | 0.66 | 0.34 | - | - |  | 0.763 | 0.237 | - | - |  |
| Leu-Cα | 0.405 | 0 | 0.595 | 0 |  | 0.349 | 0 | 0.651 | 0 |  | 0.365 | 0 | 0.625 | 0 |  |
| Leu-Cβ | 1 | 0 | - | 0 |  | 0.895 | 0.105 | - | 0 |  | 1 | 0 | - | 0 |  |
| Leu-Cδ^1^ | 0.435 | 0.565 | - | - |  | 0.37 | 0.63 | - | - |  | 0.35 | 0.65 | - | - |  |
| Leu-Cδ^2^ | 1 | 0 | - | - |  | 0.982 | 0.018 | - | - |  | 1 | 0 | - | - |  |
| Lys-Cα | 0.366 | 0 | 0.634 | 0 |  | 0.366 | 0 | 0.633 | 0 |  | 0.319 | 0 | 0.681 | 0 |  |
| Lys-Cβ | 0.754 | 0.145 | - | - |  | 0.7 | 0.294 | - | - |  | 0.769 | 0.227 | - | - |  |
| Lys-Cγ | 0.719 | 0.281 | - | - |  | 0.689 | 0.308 | - | - |  | 0.741 | 0.259 | - | - |  |
| Lys-Cδ | 0.411 | 0.589 | - | - |  | 0.32 | 0.68 | - | - |  | 0.306 | 0.694 | - | - |  |
| Lys-Cε | 0.402 | 0.598 | - | - |  | 0.328 | 0.672 | - | - |  | 0.329 | 0.671 | - | - |  |
| Met-Cα | 0.414 | 0.177 | 0.227 | 0.181 |  | 0.424 | 0.126 | 0.243 | 0.207 |  | 0.382 | 0.161 | 0.281 | 0.176 |  |
| Phe-Cα | 0.265 | 0.16 | 0.123 | 0.451 |  | 0.228 | 0.138 | 0.073 | 0.562 |  | 0.267 | 0.118 | 0.032 | 0.582 |  |
| Phe-Cβ | 0.382 | 0.618 | 0 | 0 |  | 0.305 | 0.695 | 0 | 0 |  | 0.276 | 0.724 | 0 | 0 |  |
| Pro-Cα | 0.492 | 0.202 | 0.241 | 0.065 |  | 0.391 | 0.207 | 0.286 | 0.117 |  | 0.448 | 0.195 | 0.289 | 0.067 |  |
| Pro-Cβ | 0.626 | 0.352 | - | 0.021 |  | 0.48 | 0.373 | - | 0.147 |  | 0.708 | 0.237 | - | 0.055 |  |
| Pro-Cγ | 0.407 | 0.593 | - | 0 |  | 0.302 | 0.664 | - | 0.034 |  | 0.312 | 0.688 | - | 0 |  |
| Pro-Cδ | 0.4 | 0.6 | - | - |  | 0.324 | 0.676 | - | - |  | 0.309 | 0.691 | - | - |  |
| Ser-Cα | 0.328 | 0.094 | 0.265 | 0.313 |  | 0.271 | 0.101 | 0.24 | 0.388 |  | 0.285 | 0.075 | 0.241 | 0.399 |  |
| Ser-Cβ | 0.628 | 0.372 | - | - |  | 0.546 | 0.454 | - | - |  | 0 | 1 | - | - |  |
| Thr-Cα | 0.48 | 0.155 | 0.242 | 0.123 |  | 0.417 | 0.162 | 0.28 | 0.142 |  | 0.478 | 0.129 | 0.26 | 0.134 |  |
| Thr-Cβ | 0.461 | 0.314 | 0.155 | 0.07 |  | 0.387 | 0.378 | 0.133 | 0.102 |  | 0.448 | 0.339 | 0.163 | 0.04 |  |
| Thr-Cγ^2^ | 0.689 | 0.311 | - | - |  | 0.622 | 0.378 | - | - |  | 0.63 | 0.37 | - | - |  |
| Tyr-Cα | 0.256 | 0.152 | 0.136 | 0.456 |  | 0.229 | 0.139 | 0.104 | 0.528 |  | 0.26 | 0.116 | 0.051 | 0.573 |  |
| Tyr-Cβ | 0.402 | 0.598 | - | - |  | 0.302 | 0.698 | - | - |  | 0.318 | 0.682 | - | - |  |
| Tyr-Cδ^x^ | 0.336 | 0.664 | - | 0 |  | 0.281 | 0.719 | - | 0 |  | 0.28 | 0.721 | - | 0 |  |
| Tyr-Cε^x^ | 0.56 | 0.07 | 0.108 | 0.256 |  | 0.519 | 0.062 | 0.13 | 0.289 |  | 0.519 | 0.047 | 0.108 | 0.325 |  |
| Val-Cα | 0.435 | 0 | 0.565 | 0 |  | 0.365 | 0 | 0.623 | 0.011 |  | 0.373 | 0 | 0.627 | 0 |  |
| Val-Cγ^1^ | 0.428 | 0.572 | - | - |  | 0.996 | 0 | - | - |  | 0.315 | 0.685 | - | - |  |
| Val-Cγ^2^ | 1 | 0 | - | - |  | 0.937 | 0.062 | - | - |  | 0.992 | 0.008 | - | - |  |

The first column indicates the carbon for which the ^13^C fines structure was observed. The *f*-values were calculated as described in Syzperski (1995) and are given for the chemostat cultivations of *P. pastoris* at μ= 0.09h^-1^ on glucose-methanol mixtures. Note that, for the terminal carbons, *f*^(2*)^ and *f*^(3)^ are not defined, and in case where f^(2*)^ is not given for a mid-chain carbon, the carbon-carbon scalar coupling constants are similar and the two doublets cannot be distinguished.

**Table 2 - Second series of labeling experiments**

| ***Strain*** | | | | | | | | | | | | | | |
| --- | --- | --- | --- | --- | --- | --- | --- | --- | --- | --- | --- | --- | --- | --- |
|  | X-33 control | | | |  | X-33 *ROL* 1-copy | | | |  | X-33 *ROL* 2-copy | | | |
| C atom | *f^(1)^* | *f^(2)^* | *f^(2*)^* | *f^(3)^* |  | *f^(1)^* | *f^(2)^* | *f^(2*)^* | *f^(3)^* |  | *f^(1)^* | *f^(2)^* | *f^(2*)^* | *f^(3)^* |
|  |  |  |  |  |  |  |  |  |  |  |  |  |  |  |
| Ala-Cα | 0.241 | 0.205 | 0.099 | 0.456 |  | 0.237 | 0.186 | 0.07 | 0.507 |  | 0.261 | 0.156 | 0.051 | 0.532 |
| Ala-Cβ | 0.333 | 0.667 | - | - |  | 0.334 | 0.666 | - | - |  | 0.342 | 0.658 | - | - |
| Arg-Cβ | 0.656 | 0.335 | - | 0 |  | 0.666 | 0.304 | - | 0 |  | 0.657 | 0.318 | - | 0.025 |
| Arg-Cδ | 0.403 | 0.597 | - | - |  | 0.378 | 0.622 | - | - |  | 0.372 | 0.628 | - | - |
| Asp-Cα | 0.464 | 0.167 | 0.23 | 0.139 |  | 0.404 | 0.161 | 0.278 | 0.157 |  | 0.483 | 0.108 | 0.274 | 0.135 |
| Asp-Cβ | 0.449 | 0.234 | 0.254 | 0.063 |  | 0.379 | 0.256 | 0.271 | 0.094 |  | 0.494 | 0.164 | 0.267 | 0.075 |
| Glu-Cα | 0.420 | 0.247 | 0.258 | 0.075 |  | 0.388 | 0.233 | 0.276 | 0.103 |  | 0.501 | 0.173 | 0.274 | 0.052 |
| Glu-Cβ | 0.646 | 0.354 | - | 0 |  | 0.636 | 0.364 | - | 0 |  | 0.772 | 0.229 | - | 0 |
| Glu-Cγ | 0.345 | 0 | 0.646 | 0 |  | 0.325 | 0 | 0.665 | 0 |  | 0.368 | 0 | 0.632 | 0 |
| Gly-Cα | 0.433 | 0.567 | - | - |  | 0.434 | 0.566 | - | - |  | 0.447 | 0.553 | - | - |
| His-Cα | 0.184 | 0.054 | 0.073 | 0.690 |  | 0.191 | 0.049 | 0.080 | 0.68 |  | 0.245 | 0.016 | 0.062 | 0.677 |
| His-Cβ | 0.263 | 0.451 | 0 | 0.286 |  | 0.306 | 0.426 | 0 | 0.268 |  | 0.329 | 0.465 | 0 | 0.205 |
| His-Cδ^2^ | 0.554 | 0.446 | - | - |  | 0.637 | 0.363 | - | - |  | 0.587 | 0.413 | - | - |
| Ile-Cα | 0.598 | 0 | 0.402 | 0 |  | 0.582 | 0 | 0.418 | - |  | 0.604 | 0 | 0.396 | 0 |
| Ile-Cγ^1^ | 0.708 | 0.257 | - | 0 |  | 0.658 | 0.295 | - | 0 |  | 0.699 | 0.301 | - | 0 |
| Ile-Cγ^2^ | 0.401 | 0.599 | - | - |  | 0.335 | 0.665 | - | - |  | 0.351 | 0.649 | - | - |
| Ile-Cδ | 0.744 | 0.256 | - | - |  | 0.713 | 0.287 | - | - |  | 0.694 | 0.306 | - | - |
| Leu-Cα | 0.401 | 0 | 0.599 | 0 |  | 0.369 | 0 | 0.631 | 0 |  | 0.384 | 0 | 0.616 | 0 |
| Leu-Cβ | 0.94 | 0.027 | - | 0.033 |  | 0.99 | 0 | - | 0.01 |  | 0.99 | 0 | - | 0.01 |
| Leu-Cδ^1^ | 0.449 | 0.551 | - | - |  | 0.361 | 0.639 | - | - |  | 0.381 | 0.619 | - | - |
| Leu-Cδ^2^ | 0.981 | 0.019 | - | - |  | 0.927 | 0.073 | - | - |  | 1 | 0 | - | - |
| Lys-Cα | 0.314 | 0.024 | 0.662 | 0 |  | 0.384 | 0 | 0.592 | 0.024 |  | 0.366 | 0 | 0.634 |  |
| Lys-Cβ | 0.682 | 0.273 | - | 0.045 |  | 0.385 | 0.593 | - | 0.022 |  | 0.756 | 0.199 | - | 0.018 |
| Lys-Cγ | 0.654 | 0.305 | - | 0.041 |  | 0.649 | 0.317 | - | 0.034 |  | 0.71 | 0.29 | - | 0 |
| Lys-Cδ | 0.397 | 0.603 | - | - |  | 0.362 | 0.044 | 0.594 | 0 |  | 0.37 | 0.017 | 0.613 | - |
| Lys-Cε | 0.401 | 0.599 | - | - |  | 0.37 | 0.63 | - | - |  | 0.373 | 0.627 | - | - |
| Met-Cα | 0.403 | 0.119 | 0.256 | 0.222 |  | 0.43 | 0.128 | 0.243 | 0.199 |  | 0.45 | 0.098 | 0.291 | 0.162 |
| Phe-Cα | 0.237 | 0.162 | 0.143 | 0.458 |  | 0.257 | 0.129 | 0.105 | 0.51 |  | 0.282 | 0.11 | 0.067 | 0.54 |
| Phe-Cβ | 0.378 | 0.622 | 0 | 0 |  | 0.338 | 0.662 | 0 | 0 |  | 0.351 | 0.649 | 0 | 0 |
| Pro-Cα | 0.498 | 0.236 | 0.216 | 0.050 |  | 0.437 | 0.206 | 0.248 | 0.135 |  | 0.499 | 0.166 | 0.262 | 0.074 |
| Pro-Cβ | 0.668 | 0.33 | - | 0.002 |  | 0.656 | 0.344 | - | 0 |  | 0.744 | 0.256 | - | 0 |
| Pro-Cγ | 0.408 | 0.592 | - | 0 |  | 0.372 | 0.617 | - | 0.011 |  | 0.374 | 0.616 | - | 0 |
| Pro-Cδ | 0.405 | 0.595 | - | - |  | 0.367 | 0.633 | - | - |  | 0.377 | 0.623 | - | - |
| Ser-Cα | 0.274 | 0.116 | 0.254 | 0.356 |  | 0.268 | 0.094 | 0.262 | 0.376 |  | 0.333 | 0.072 | 0.236 | 0.352 |
| Ser-Cβ | 0.559 | 0.441 | - | - |  | 0.557 | 0.443 | - | - |  | 0.586 | 0.414 | - | - |
| Thr-Cα | 0.471 | 0.142 | 0.253 | 0.135 |  | 0.443 | 0.176 | 0.238 | 0.143 |  | 0.502 | 0.116 | 0.271 | 0.11 |
| Thr-Cβ | 0.449 | 0.156 | 0.33 | 0.066 |  | 0.409 | 0.35 | 0.15 | 0.091 |  | 0.489 | 0.306 | 0.146 | 0.059 |
| Thr-Cγ^2^ | 0.654 | 0.346 | - | - |  | 0.582 | 0.418 | - | - |  | 0.673 | 0.327 | - | - |
| Tyr-Cα | 0.236 | 0.177 | 0.134 | 0.452 |  | 0.22 | 0.145 | 0.093 | 0.542 |  | 0.288 | 0.12 | 0.0721 | 0.521 |
| Tyr-Cβ | 0.394 | 0.606 | 0 | 0 |  | 0.36 | 0.64 | 0 | 0 |  | 0.347 | 0.653 | 0 | 0 |
| Tyr-Cδ^x^ | 0.339 | 0.661 | - | 0 |  | 0.259 | 0.672 | - | 0.04 |  | 0.309 | 0.691 | - | 0 |
| Tyr-Cε^x^ | 0.469 | 0.071 | 0.139 | 0.321 |  | 0.523 | 0.056 | 0.136 | 0.285 |  | 0.586 | 0.03 | 0.107 | 0.277 |
| Val-Cα | 0.415 | 0 | 0.585 | 0 |  | 0.384 | 0 | 0.616 | 0 |  | 0.393 | 0 | 0.607 | 0 |
| Val-Cγ^1^ | 0.405 | 0.595 | - | - |  | 0.374 | 0.626 | - | - |  | 0.408 | 0.592 | - | - |
| Val-Cγ^2^ | 0.947 | 0.053 | - | - |  | 1 | 0 | - | - |  | 0.978 | 0.022 | - | - |

The first column indicates the carbon for which the ^13^C fines structure was observed. The *f*-values were calculated as described in Szyperski (1995) and are given for the chemostat cultivations of *P. pastoris* at μ= 0.09h^-1^ on glucose-methanol mixtures. Note that, for the terminal carbons, *f*^(2*)^ and *f*^(3)^ are not defined, and in case where f^(2*)^ is not given for a mid-chain carbon, the carbon-carbon scalar coupling constants are similar and the two doublets cannot be distinguished.
