# Supplementary material for: Metabolic flux profiling of recombinant protein secreting Pichia pastoris growing on glucose:methanol mixtures
Source: Microb Cell Fact. 2012 May 8;11:57. doi: 10.1186/1475-2859-11-57 (PMC3443025; doi:10.1186/1475-2859-11-57)
Supplement: Additional file 3 — Metabolic flux distributions in the P. pastoris reference and recombinant strains during growth on glucose:methanol. Metabolic flux distributions in the P. pastoris reference strain (top), the recombinant strain with 1 copy of the ROL gene (middle) and the recombinant strain harbouring 2 copies of the ROL gene (bottom) during growth on glucose:methanol chemostat cultures at about 0.09 h−1. Activities of the malic enzyme and glyoxylate pathways were found to be negligible on the basis of the METAFoR analyses. [file 1475-2859-11-57-S3.ppt]

## Slide 1
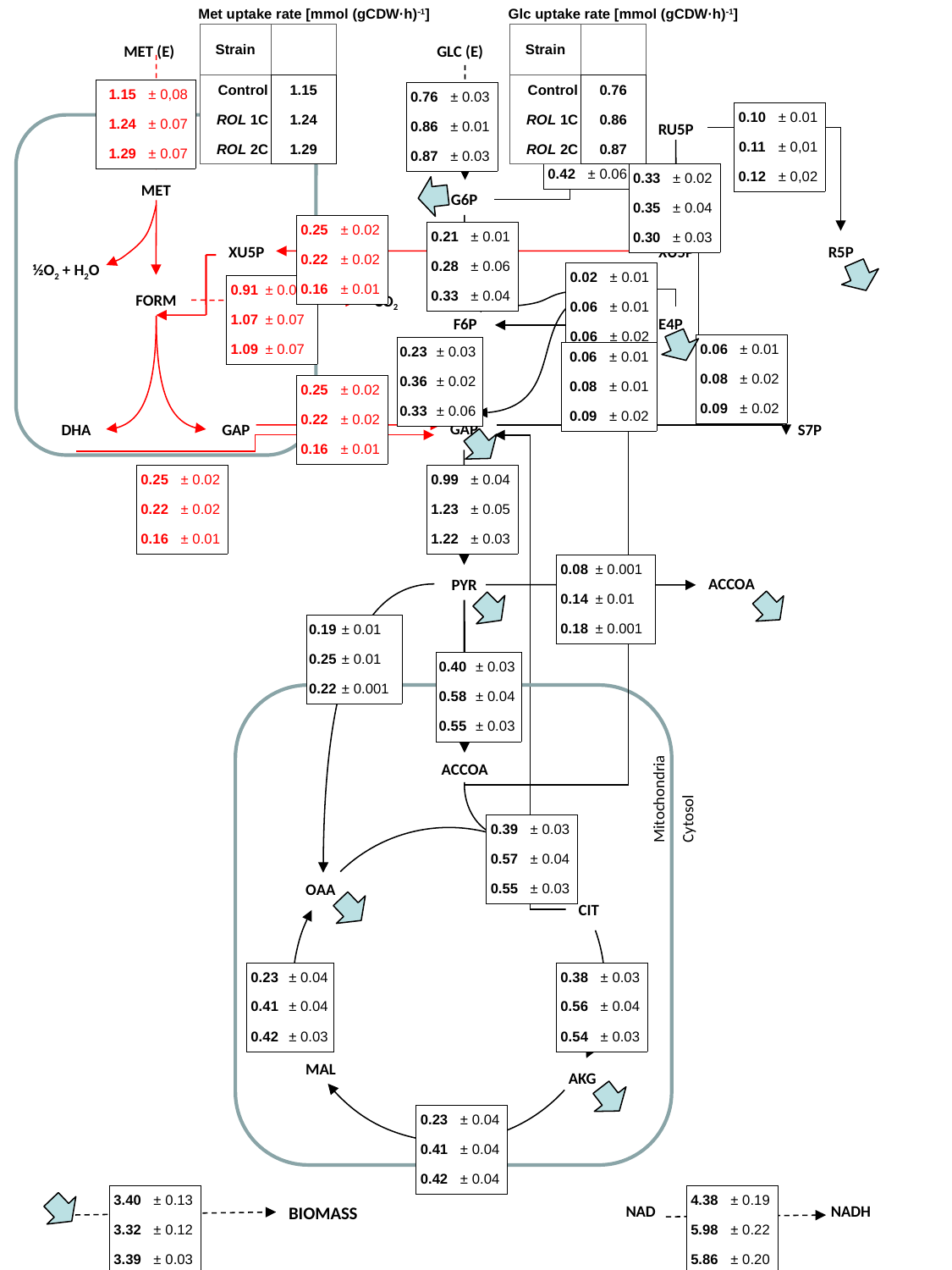

Met uptake rate [mmol (gCDW·h)−1]
Glc uptake rate [mmol (gCDW·h)−1]
| Strain | |
| --- | --- |
| Control | 1.15 |
| ROL 1C | 1.24 |
| ROL 2C | 1.29 |
| Strain | |
| --- | --- |
| Control | 0.76 |
| ROL 1C | 0.86 |
| ROL 2C | 0.87 |
MET (E)
GLC (E)
| 1.15 | ± 0,08 |
| --- | --- |
| 1.24 | ± 0.07 |
| 1.29 | ± 0.07 |
| 0.76 | ± 0.03 |
| --- | --- |
| 0.86 | ± 0.01 |
| 0.87 | ± 0.03 |
| 0.43 | ± 0.03 |
| --- | --- |
| 0.45 | ± 0.04 |
| 0.42 | ± 0.06 |
| 0.10 | ± 0.01 |
| --- | --- |
| 0.11 | ± 0,01 |
| 0.12 | ± 0,02 |
RU5P
| 0.33 | ± 0.02 |
| --- | --- |
| 0.35 | ± 0.04 |
| 0.30 | ± 0.03 |
MET
G6P
| 0.25 | ± 0.02 |
| --- | --- |
| 0.22 | ± 0.02 |
| 0.16 | ± 0.01 |
| 0.21 | ± 0.01 |
| --- | --- |
| 0.28 | ± 0.06 |
| 0.33 | ± 0.04 |
XU5P
XU5P
R5P
½O2 + H2O
| 0.02 | ± 0.01 |
| --- | --- |
| 0.06 | ± 0.01 |
| 0.06 | ± 0.02 |
| 0.91 | ± 0.06 |
| --- | --- |
| 1.07 | ± 0.07 |
| 1.09 | ± 0.07 |
FORM
CO2
F6P
E4P
| 0.06 | ± 0.01 |
| --- | --- |
| 0.08 | ± 0.02 |
| 0.09 | ± 0.02 |
| 0.23 | ± 0.03 |
| --- | --- |
| 0.36 | ± 0.02 |
| 0.33 | ± 0.06 |
| 0.06 | ± 0.01 |
| --- | --- |
| 0.08 | ± 0.01 |
| 0.09 | ± 0.02 |
| 0.25 | ± 0.02 |
| --- | --- |
| 0.22 | ± 0.02 |
| 0.16 | ± 0.01 |
GAP
DHA
GAP
S7P
| 0.25 | ± 0.02 |
| --- | --- |
| 0.22 | ± 0.02 |
| 0.16 | ± 0.01 |
| 0.99 | ± 0.04 |
| --- | --- |
| 1.23 | ± 0.05 |
| 1.22 | ± 0.03 |
| 0.08 | ± 0.001 |
| --- | --- |
| 0.14 | ± 0.01 |
| 0.18 | ± 0.001 |
ACCOA
PYR
| 0.19 | ± 0.01 |
| --- | --- |
| 0.25 | ± 0.01 |
| 0.22 | ± 0.001 |
| 0.40 | ± 0.03 |
| --- | --- |
| 0.58 | ± 0.04 |
| 0.55 | ± 0.03 |
ACCOA
Mitochondria
Cytosol
| 0.39 | ± 0.03 |
| --- | --- |
| 0.57 | ± 0.04 |
| 0.55 | ± 0.03 |
OAA
CIT
| 0.23 | ± 0.04 |
| --- | --- |
| 0.41 | ± 0.04 |
| 0.42 | ± 0.03 |
| 0.38 | ± 0.03 |
| --- | --- |
| 0.56 | ± 0.04 |
| 0.54 | ± 0.03 |
MAL
AKG
| 0.23 | ± 0.04 |
| --- | --- |
| 0.41 | ± 0.04 |
| 0.42 | ± 0.04 |
| 3.40 | ± 0.13 |
| --- | --- |
| 3.32 | ± 0.12 |
| 3.39 | ± 0.03 |
| 4.38 | ± 0.19 |
| --- | --- |
| 5.98 | ± 0.22 |
| 5.86 | ± 0.20 |
BIOMASS
NAD
NADH
