# Supplementary material for: Metabolic flux profiling of recombinant protein secreting Pichia pastoris growing on glucose:methanol mixtures
Source: Microb Cell Fact. 2012 May 8;11:57. doi: 10.1186/1475-2859-11-57 (PMC3443025; doi:10.1186/1475-2859-11-57)
Supplement: Additional file 4 — Stoichiometric model of the central carbon metabolism of P. pastoris . Reactions in the stoichiometric model of the central carbon metabolism of P. pastoris applied in the 13C-MFA determination of the metabolic fluxes under different oxygenation conditions; it also includes anabolic reactions from metabolic intermediates to biosynthesis, transport reactions across the mitochondrial membrane and uptake and excretion reactions. Note that O2, CO2, energy and redox cofactor mass balances were not included in the mass balance constraints in 13C-MFA. [file 1475-2859-11-57-S4.doc]

**Supplementary file 4. Stochiometric model for *P. pastoris***

**Methanol Metabolism**

1. MET => FORM
2. FORM => FOR + NADH
3. FOR => NADH + CO_2_
4. XU5P + FORM + ATP => ADP + GAP + DHA
5. GAP => G3P
6. DHA => G3P

**Glycolysis and Gluconeogenesis Pathways**

1. GLC + ATP => G6P + ADP
2. G6P <=> F6P
3. F6P + ATP => 2G3P + ADP
4. 2 G3P => F6P +Pi
5. G3P + ADP + Pi => 3PG + ATP + NADH
6. 3PG + ATP + NADH => G3P + ADP + Pi
7. 3PG <=> Pep
8. Pep + ADP => Pyr + ATP
9. Pyr => ACCoA_mit_ + CO_2_ + NADH_mit_
10. Pyr => ACCoA_cyt_ +CO_2_ + NADH_cyt_
11. Pyr + CO_2_ + ADP => Oaa + ATP

**Pentose Phosphate Pathway**

1. G6P + 2NADP => RU5P + 2 NADPH + CO_2_
2. RU5P <=> R5P
3. RU5P <=> XU5P
4. R5P + XU5P <=> S7P + G3P
5. S7P + G3P <=> F6P + E4P
6. XU5P + E4P <=> F6P + G3P

**TCA cycle**

1. ACCoA_mit_ + Oaa => CIT
2. CIT => ICIT
3. ICIT => AKG + CO_2_ + NADH_mit_
4. AKG => SUCCoA + CO_2_ + NADH_mit_
5. SUCCoA + Pi + ADP => SUC + ATP
6. SUC + ATP => SUCCoA + ADP + Pi
7. SUC => FUM + FADH_2_
8. FUM <=> MAL
9. MAL => Oaa + NADH_mit_

**Biosynthesis of amino acids**

**Serine Family**

1. 3PG + Glu => Ser + AKG + NADH +Pi
2. Ser + THF => Gly + MetTHF
3. Ser + ACCoA + H_2_S => Cys

**Alanine Family**

1. Pyr + NADPH => Ala + NADP
2. Pyr + Glu => AKG
3. 2 Pyr + NADPH_mit_ => Kval + CO_2_
4. Kval + Glu => Val + AKG
5. Kval + ACCoA_mit_ + Glu => Leu + AKG + NADH + CO_2_

**Histidine Family**

1. R5P + ATP => PRPP + AMP
2. PRPP + ATP + Gln => His + AKG + Pi + 2 NADH

**Aspartic Family**

1. OAA + Glu => Asp + AKG
2. Asp + Gln + ATP => Asn + Glu + AMP
3. Asp + ATP + 2 NADPH => Ser + ADP + Pi
4. Thr + NADPH_mit_ + Glu + Pyr => Ile + AKG + NH_4_ + CO_2_
5. ACCoA + Ser + H_2_S + MTHF => Met + THF

**Aromatic Family**

1. 2 Pep + E4P + ATP + NADPH => CHOR + ADP + 4 Pi
2. CHOR + Glu => Phe + AKG + CO_2_
3. CHOR + Glu => Tyr + AKG + NADH + CO_2_
4. CHOR + Gln + PRPP + Ser => Trp + Glu + Pyr + G3P + CO2

**Glutamic Family**

1. AKG +NH_4_ + NADPH => Glu
2. Glu + ATP + NH_4_ => Gln + ADP + Pi
3. Glu + ATP + 2 NADPH => Pro + ADP + Pi
4. Gln + CO_2_ + 2 ATP => CaP + Glu + 2 ADP + Pi
5. Glu + ACCoA + 4 ATP + NADPH_mit_ + CaP + Asp => Arg + AKG + 4 ADP + FUM + 5 Pi
6. 2 Glu + ACCoA + 3 ATP + 2 NADPH_mit_ => Lys + AKG + CO_2_ + 2 NADH

**Biosynthesis and Interconversion of One-carbon Units**

1. DHF + NADPH => THF
2. Gly + THF => MetTHF + NH_4_ + NADH + CO_2_
3. MetTHF + NADH => THF

**Transport Reactions**

1. CO_2_ => exp
2. imp => CO_2_
3. imp => NH_4_
4. NH_4_ => exp
5. imp => Pi
6. Pi => exp
7. NAD => NADH_mit_

**Biomass Synthesis**

**Protein synthesis**

*X-33 control strain*

1. 0.1488 Pyr + 0.00725 R5P + 0.0147 E4P + 0.0633 AKG_mit_ + 0.044 Oaa_mit_ + 0.0137 ACCoA_cyt_ => 1 C-mol Protein + 0.004 G3P + 0.0448 CO_2_

*X-33 ROL 1-copy strain*

1. 0.1419 Pyr + 0.0068 R5P + 0.014 E4P + 0.0419 Oaa_mit_ + 0.0681 AKG_cyt_ + 0.0136 ACCoA_cyt_ => 1 C-mol Protein + 0.0029 G3P + 0.0445 CO_2_

*X-33 ROL 2-copy strain*

1. 0.1491 Pyr + 0.00699 R5P + 0.0153 E4P + 0.044 Oaa_mit_ + 0.062 AKG_cyt_ + 0.01454 ACCoA_cyt_ => 1 C-mol Protein + 0.0031 G3P + 0.0502 CO_2_

**Carbohydrate Synthesis**

1. 0.113 G6P + 0.053 F6P => 1 C-mol Carbohydrate

**Lipids Synthesis**

1. 0.002 G6P + 0.0055 Pyr + 0.011 G3P + 0.006 CO_2_ + 0.039 ACCoA_mit_ + 0.441 ACCoA_cyt_ + 0.065 O_2_ => 1 C-mol Lipid

**RNA Synthesis**

1. 0.056 Pyr + 0.1136 CO_2_ + 0.105 R5P + 0.0479 Oaa_mit_ => 1 C-mol RNA

**DNA Synthesis**

1. 0.051 Pyr + 0.132 CO_2_ + 0.102 R5P + 0.051 Oaa_mit_ => 1 C-mol DNA

**ROL Synthesis**

1. 0.171 Pyr + 0.0067 R5P + 0.022 E4P + 0.0472 Oaa_mit_ + 0.029 AKG_cyt_ + 0.0164 AKG_mit_ + 0.0112 ACCoA_cyt_ => 1 C-mol Protein + 0.0022 G3P + 0.0689 CO_2_
